# Inflammation and its role in age-related macular degeneration

**DOI:** 10.1007/s00018-016-2147-8

**Published:** 2016-02-06

**Authors:** Anu Kauppinen, Jussi J. Paterno, Janusz Blasiak, Antero Salminen, Kai Kaarniranta

**Affiliations:** Faculty of Health Sciences, School of Pharmacy, University of Eastern Finland, P.O. Box 1627, 70211 Kuopio, Finland; Department of Ophthalmology, Kuopio University Hospital, Kuopio, Finland; Department of Ophthalmology, Institute of Clinical Medicine, University of Eastern Finland, Kuopio, Finland; Department of Molecular Genetics, Faculty of Biology and Environmental Protection, University of Lodz, Lodz, Poland; Department of Neurology, Institute of Clinical Medicine, University of Eastern Finland, Kuopio, Finland

**Keywords:** Signaling, Aging, Immune system, NLRP3, Eye, Retina

## Abstract

Inflammation is a cellular response to factors that challenge the homeostasis of cells and tissues. Cell-associated and soluble pattern-recognition receptors, e.g. Toll-like receptors, inflammasome receptors, and complement components initiate complex cellular cascades by recognizing or sensing different pathogen and damage-associated molecular patterns, respectively. Cytokines and chemokines represent alarm messages for leukocytes and once activated, these cells travel long distances to targeted inflamed tissues. Although it is a crucial survival mechanism, prolonged inflammation is detrimental and participates in numerous chronic age-related diseases. This article will review the onset of inflammation and link its functions to the pathogenesis of age-related macular degeneration (AMD), which is the leading cause of severe vision loss in aged individuals in the developed countries. In this progressive disease, degeneration of the retinal pigment epithelium (RPE) results in the death of photoreceptors, leading to a loss of central vision. The RPE is prone to oxidative stress, a factor that together with deteriorating functionality, e.g. decreased intracellular recycling and degradation due to attenuated heterophagy/autophagy, induces inflammation. In the early phases, accumulation of intracellular lipofuscin in the RPE and extracellular drusen between RPE cells and Bruch’s membrane can be clinically detected. Subsequently, in dry (atrophic) AMD there is geographic atrophy with discrete areas of RPE loss whereas in the wet (exudative) form there is neovascularization penetrating from the choroid to retinal layers. Elevations in levels of local and systemic biomarkers indicate that chronic inflammation is involved in the pathogenesis of both disease forms.

## Introduction

### Overview of inflammation

Inflammation is a rapid response mounted by the cell to a threat of imminent danger. Inflammation is intended to eliminate foreign or damaged material, and to signal to other cells that there is a danger in order that they can initiate a broader immune response. Later, it should initiate tissue recovery. Monocyte-derived phagocytizing innate immune cells of myeloid origin which are present in many tissues, such as macrophages, microglia, and Kupffer cells, play a key role in the initiation of inflammation and recently, the capacity of granulocytes to initiate inflammation has been recognized [1]. Also other cells are involved in the induction of inflammation; e.g. epithelial cells which until recently were thought mainly to provide a mechanical barrier [2]. Inflammation can be induced by a wide variety of signals, ranging from microbes and other foreign material to mechanical tissue injury and autoantigens. A threat becomes recognized by pattern-recognition receptors (PRRs). From the short-term point of view, inflammation is highly advantageous, e.g. when it is a response to microbial infection or mechanical injuries, but long-term inflammation is detrimental. Prolonged low-level inflammation has been linked with the development of various chronic conditions, such as cancer, diabetes, autoimmune diseases, as well as several obesity-related and neurodegenerative diseases [3, 4].

### Age-related macular degeneration (AMD)

**Fig. 1:**
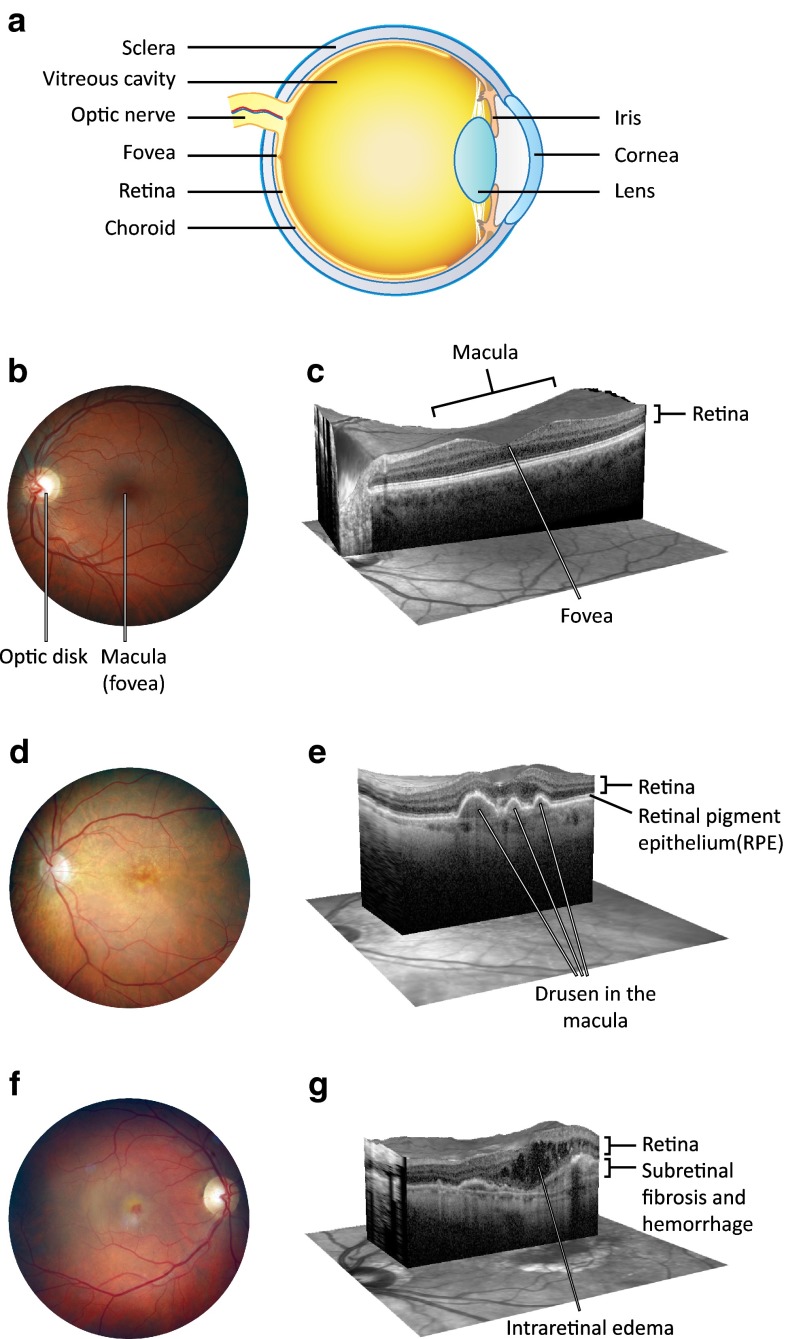
**a** A schematic transverse section through the human eyeball. The macula is located in the posterior pole of the eye. In the center of the macula, a shallow depression in the retina (the fovea) marks the area with the highest visual acuity. Light enters the eye and bends to the sensory retina in the fovea by passing through the transparent media including cornea, lens, and the vitreous body. The sensory retina converts light into nerve impulses, processes the information, and sends it along the visual pathway to the visual cortex. **b** A normal human retina. A* colored photograph* of the fundus from the left eye of a healthy subject. The macula is located in the center of the retina. **c** A cross-section of the normal macula. An OCT scan through the fovea of the healthy left eye reveals the normal organization of the retinal layers. Normal anatomy of the fovea is important for accurate central vision. Modern OCT is an important in vivo tool for ophthalmologists since it allows them to monitor different pathologies non-invasively in this important but tiny and cell-dense location. **d** A fundus photograph from the left eye of an individual with dry AMD. This demonstrates the presence of numerous yellow deposits, known as drusen, in the central macula. **e** A cross-section of the macula from an individual with dry AMD. The OCT scan through the fovea of the left eye shows three drusen under the RPE layer. This eye would be expected to suffer from image distortion, as central drusen are prone to reshape the normal foveal pit. Large drusen are associated with decreased visual acuity and disruption of energy homeostasis in the retina. **f** A fundus photograph from the right eye of an individual with wet AMD. Significant macular edema and exudates together with foveal hemorrhage occur but only small sparse drusen are present centrally. **g** A cross-section from the macula in the right eye of an individual with wet AMD. An OCT scan through the location of the fovea shows the formation of intraretinal fluid cysts in the fovea. Edema causes the foveal pit to disappear. The local retinal swelling in wet AMD is due to the leaky, abnormal vessels sprouting from the underlying choroid. Intraretinal edema disrupts the normal retinal layer organization and leads to a retinal dysfunction. The OCT scan reveals also a potential hemorrhage and fibrotic lesion development in the fovea. This is another typical finding in wet AMD, likely to result in a permanent central visual field loss, if left untreated. *AMD* age-related macular degeneration, *OCT* optical coherence tomography, *RPE* retinal pigment epithelium

AMD is a progressive eye disease that has been linked with several pathological factors, i.e. chronic oxidative stress, autophagy decline, and inflammation [5–10]. It is the most common reason for irreversible vision impairment in aged individuals in the developed countries where refractive errors, cataract, and glaucoma are now efficiently treated. Early AMD is usually asymptomatic, although retinal pigment epithelium (RPE) mottling and extracellular drusen deposits between RPE cells and Bruch’s membrane can be clinically detected in the central posterior pole of the eye [6] (Fig. 1). Bruch’s membrane is a five-layered sheet lying over the highly vascularized choroid, and it makes contacts with both vascular endothelium and RPE. The accumulation of drusen increases an individual’s risk of developing advanced AMD. AMD is subdivided into two types, dry and wet AMD forms, also known as geographic atrophy and exudative AMD, respectively (Fig. 1). In wet AMD, the RPE produces excessive amounts of vascular endothelial growth factor (VEGF), and this contributes to the breakdown of the blood-retinal barrier and sprouting of fragile blood vessels from the choroid through Bruch’s membrane into the retina in a process called neovascularization. Leakage of blood from these abnormal vessels causes oedema and an acute loss of vision [11, 12]. As the world’s population ages, the global burden of AMD will increase, posing a huge burden on the health care system [13–15]. Therefore, efforts have been made to resolve the pathophysiology of AMD and to develop effective treatments. During the recent decade, the management of the wet AMD has advanced dramatically due to the arrival of anti-VEGF therapies [16]. Currently, there are several different forms of effective intravitreal treatment available for decelerating the progress of wet AMD but unfortunately no such advances have been made in the treatment of dry AMD, the disease type that accounts for the majority (up to 90 %) of cases [17–20]. A reduction of intracellular inflammation in conjunction with the prevention of RPE and photoreceptor loss all have central roles in programmes developing novel therapy options for AMD [21].

### Retinal pigment epithelium in the pathogenesis of AMD

The RPE, a single-cell layer at the posterior part of the eye plays a significant role in the pathogenesis of AMD. RPE cells are responsible for many tasks in the eye including maintaining the functionality of the overlying photoreceptor cells, protection of the retina from excessive light, formation of blood-retinal barrier in conjunction with the vascular endothelium, and immune defence of the central retina (macula) [22, 23]. A functional degeneration of the RPE results in impaired maintenance of sensory retina, which contributes to the vision loss in advanced AMD. The photoreceptors most severely affected are located in the macular area, which is responsible for the accurate vision and colour detection and therefore AMD greatly impairs the ability of an elderly patient to lead an independent life [6, 24]. In addition, scotomas developing in the central vision field also distort the ability to see pictures, e.g. causing a disturbance in reading, dialing numbers and facial recognition.

Due to its high metabolic activity and the associated abundant oxygen consumption, its high contents of polyunsaturated fatty acids and substantial exposure to light, the RPE is especially sensitive to excessive oxidative stress [25, 26]. One of the major functions of RPE is the autophagic degradation of spent tips of photoreceptor outer segments (POS) in a process called heterophagy [22, 25]. Continuous ingestion of POS material by non-dividing and aging RPE cells results in the accumulation of an undegradable and autofluorescent metabolite called lipofuscin in lysosomes, which inhibits autophagy by blocking the function of lysosomal enzymes, i.e. it combines oxidative stress with retinal inflammation [10, 25, 27].

## Pattern-recognition receptors

Cells recognize various endogenous and exogenous pathogen- and damage-associated molecular patterns (PAMPs and DAMPs, respectively) through their evolutionarily conserved pathogen recognition receptors (PRRs) [28]. There are several cell-associated PRRs, e.g. Toll-like receptors (TLRs), receptor for advanced glycation end products (RAGE) [29–32], NOD-like receptors [NLRs; nucleotide-binding domain, leucine-rich repeat-containing (NBD-LRR) proteins], C-type lectin receptors (CLRs), retinoic acid-inducible gene (RIG)-I-like receptors (RLRs), and cytosolic DNA sensors [28, 33, 34]. Once the receptor is activated by its ligand, it rapidly induces the activation of complex intracellular signaling pathways to produce pro-inflammatory mediators [34]. PRR signaling also induces the expression of co-stimulatory molecules (e.g. CD40, CD80, or CD86) contributing to the conversion of different types of T cells, and promoting the activation of dendritic cells such that they become capable of presenting foreign peptides to lymphocytes and trigger the activation of adaptive immunity [35].

### TLRs

**Fig. 2:**
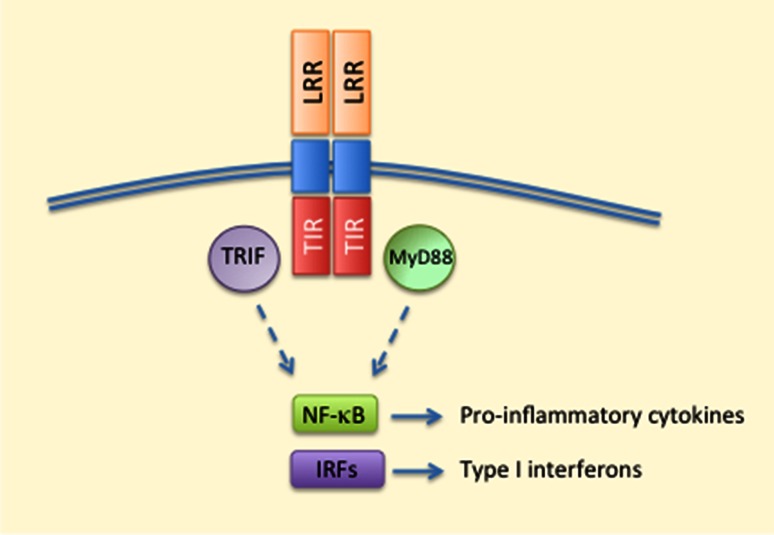
Major aspects of TLR signaling. Ligand recognition by LRR domains triggers the dimerisation of TLR proteins. MyD88 and TRIF are the principal adaptor proteins interacting with intracellular TIR domains and mediating the activation of transcription factors, such as NF-κB and IRFs for the production of pro-inflammatory cytokines and type I interferons. *IRFs* interferon regulatory factors, *LRR* leucine-rich repeat, *MyD88* myeloid differentiation-primary response gene 88, *NF-κB* nuclear factor kappa B, *TIR* Toll/IL-1 receptor domain, *TRIF* TIR-domain-containing adaptor inducing IFN-β

TLRs were the first PRRs to be discovered in the mid-1990s [33]. These are transmembrane proteins capable of recognizing a multitude of extra- and intracellular pathogens [33]. TLRs 1, 2, 4, 5, 6, and 10 are expressed on the cell surface, whereas TLRs 3, 7, 8, and 9 reside inside the cell [34, 36]. TLRs contain a ligand-sensing leucine-rich repeat (LRR) domain, a transmembrane domain, and a cytoplasmic Toll/IL-1 receptor (TIR) domain, and the receptors function as either homo- or heterodimers [33, 36] (Fig. 2). Roughly, TLR signaling can be divided into MyD88-dependent pathways that result in the production of pro-inflammatory cytokines, and TRIF-dependent signaling that aims at producing type I interferons in response to viral infections [33] (Fig. 2). Although TLR signaling results in the secretion of inflammatory cytokines, chemokines, type I interferons, and antimicrobial peptides, its crosstalk with other PRRs, such as membrane-bound CLRs, and cytosolic NLRs and RLRs, is important in the regulation of immune responses. One good example of PRR collaboration is the activation of inflammasome signaling. TLR signaling produces the pro-forms of inflammasome-dependent cytokines IL-1β and IL-18, which remain inactive in the cytoplasm until a second signal is sensed, e.g. by NLRs, leading to the maturation and secretion of these cytokines after inflammasome assembly and caspase-1-mediated proteolysis [37].

### RAGE

RAGE (receptor for advanced glycation end products) has been compared to TLRs because of its presence on the plasma membrane as well as its pro-inflammatory functions mediated through NF-κB signaling [31]. As a member of immunoglobulin superfamily, it also promotes leukocyte recruitment to inflamed tissue by functioning as an endothelial adhesion receptor [29, 30]. Originally, RAGE was considered to be a receptor for advanced glycation end products (AGEs), non-enzymatically glycated or oxidated biomolecules [29–31]. Subsequently, also other ligands, such as those released from dying cells or injured tissue, have been observed to be recognized by RAGE [32]. Those factors include the normally nuclear high mobility group box 1 protein (HMGB1) and calcium-binding S100 proteins. Interestingly, RAGE can also be activated by β-amyloid, which is a compound closely associated with the development of neurodegenerative disorders, such as Alzheimer’s disease and AMD [32, 38].

### CLRs

C-type lectin receptors (CLRs) are calcium-dependent PRRs that were originally thought to respond only to carbohydrates [39]. Nowadays, the CLR family also includes proteins that do not necessarily sense carbohydrates, but contain one or more domains homologous to the carbohydrate recognition domains of traditional CLRs. CLRs can be divided into two types of cell-associated and one type of soluble receptors [39]. The transmembrane receptors can be further divided into group I and group II CLRs that belong to the mannose and asialoglycoprotein receptor families, respectively. The activation of CLRs induces complex intracellular signaling cascades and can interact with processes mediated by other PRRs.

### NLR

**Fig. 3:**
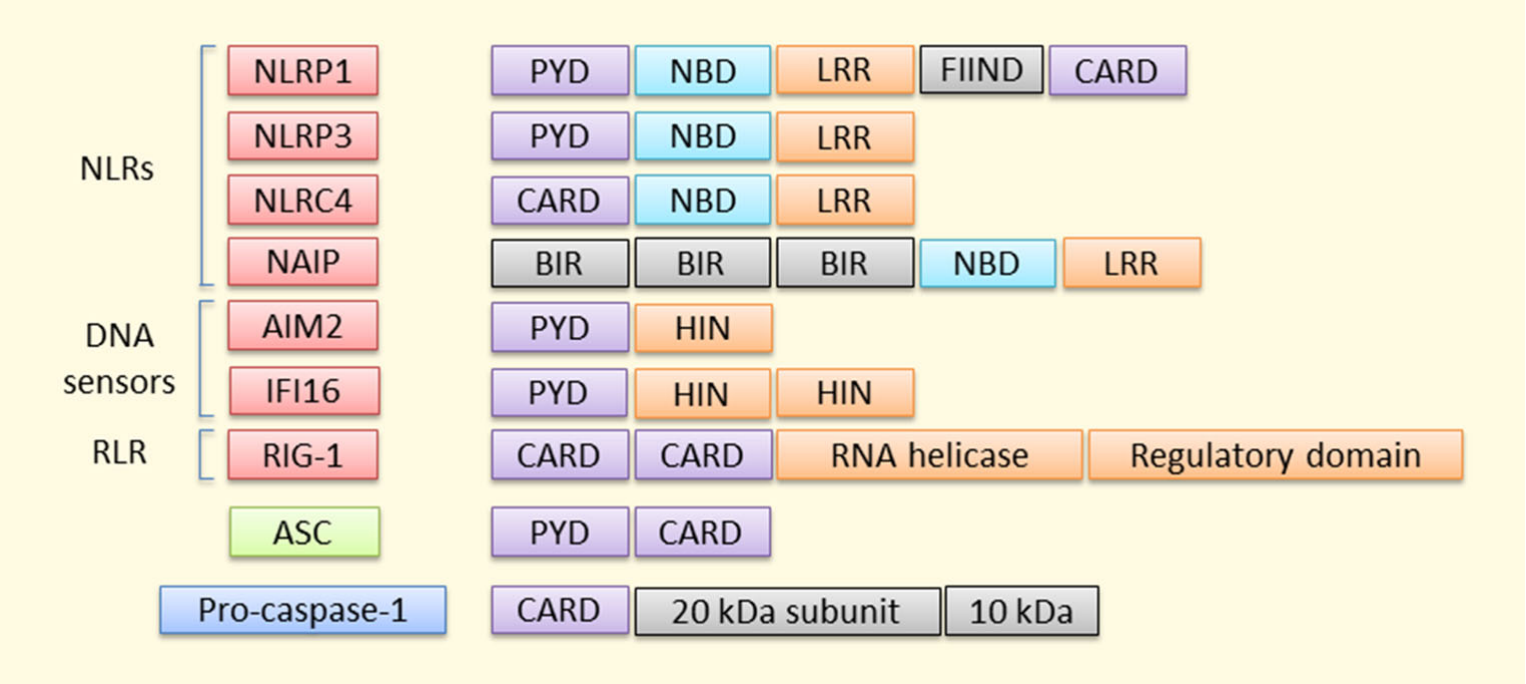
Pro-inflammatory inflammasomes. Four NLRs, two DNA sensors, and an RLR are currently the most well-known inflammasomes that promote inflammation by resulting in the release of inflammatory mediators. Receptors lacking the CARD domain are dependent on the adaptor protein ASC for their interaction with pro-caspase 1, which becomes activated by autocleavage into 20 and 10 kDa subunits by the complex assembly

To date, at least 23 human NLRs have been identified [40]. NLR proteins can be divided into four subfamilies according to their N-terminal domains. These receptors commonly contain three main domains: (1) N-terminal acidic transactivation domain (NLRA proteins), baculoviral inhibitory repeat (BIR)-like domain (NLRB proteins), caspase recruitment domain (CARD; NLRC proteins), or pyrin domain (PYD; NLRP proteins) that either recruit adaptor, intermediary, or effector components for downstream signaling; (2) central NBD (nucleotide-binding domain) or NACHT (NAIP, CIITA, HET-E, and TP1) domain that is responsible for the activation-induced oligomerization; (3) the ligand-sensing C-terminal LRR (leucine-rich repeat) domain [41]. There is an evidence suggesting that at least nine human NLRs (NLRP 1, 2, 3, 6, 7, 12, NLRC4, NAIP5, and NOD2) are able to regulate caspase-1 activation and IL-1β/IL-18 processing [40, 42–48], and NLRP1, NLRP3, NLRC4, and NAIP5 have been associated with the inflammasome activation in a broader sense (Fig. 3). Other NLRs exert variable functions, e.g. regulation of antigen presentation (NLRC5 and CIITA), inhibition/modulation of inflammation (NLRC3, NLRPs 6 and 12, NLRX1), and embryonic development (NLRPs 2, 5, and 7) [49].

#### NLRP3

**Fig. 4:**
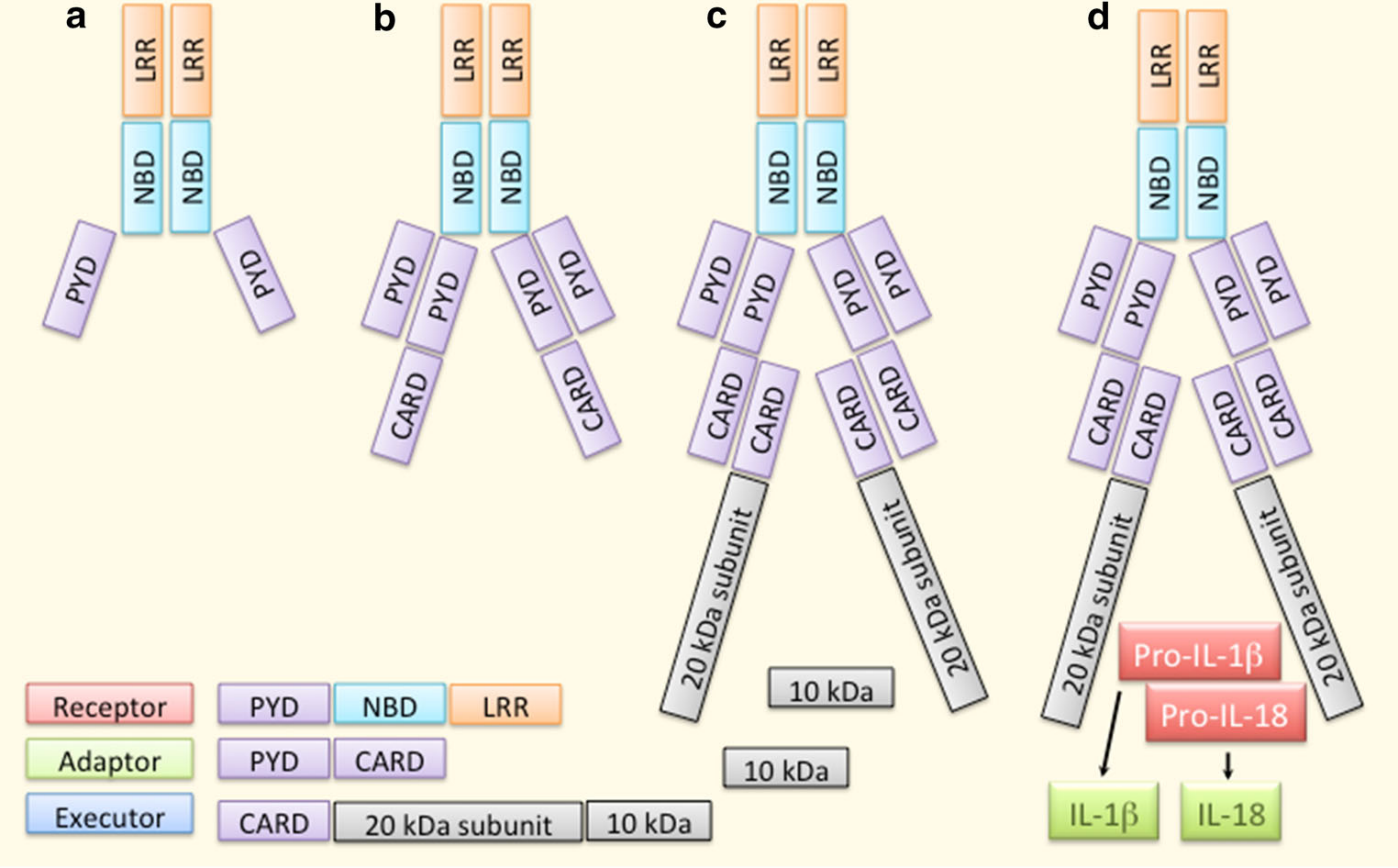
Overview of the NLRP3 inflammasome. Ligand recognition through LRR domains results in a conformational change and oligomerization of NLRP3 receptor proteins (**a**). Thereafter, the adaptor protein ASC binds NLRP3 by PYD–PYD interactions (**b**). Binding of pro-caspase-1 to ASC through CASP–CASP interactions promotes autocleavage and thereby activation of the caspase-1 enzyme (**c**). Finally, caspase-1 cleaves the pro-inflammatory cytokines IL-1β and IL-18 into their mature and secreted forms (**d**)

At present, NLRP3 [NALP3, cryopyrin, caterpillar-like receptor 1.1 (CLR1.1), CIAS1, PYPAF1] is the best-characterized inflammasome receptor (Fig. 4). In resting cells, its expression is low at both the mRNA and protein levels, but it is induced by several priming signals, e.g. mediated by TLRs, NODs, or cytokine receptors [50]. NLRP3 has a versatile recognition capacity in that it can sense both endogenous and exogenous factors and these can be biological, chemical, or physical in their nature [37, 51]. Following the ligand sensing, NLRP3 protein changes its conformation and becomes oligomerized (Fig. 4). Thereafter, pro-caspase-1 binds the complex through the adaptor protein ASC [52, 53]. The assembly of the active inflammasome results in the auto-activation of caspase-1, which subsequently cleaves the pro-inflammatory cytokines IL-1β and IL-18 into their mature and secreted forms (Fig. 4).

### Other inflammasome-related PRRs

When it was observed that all cytosolic DNA does not become sensed by NLRP3, additional DNA sensors were sought. In 2009, four research groups arrived at the same conclusion, i.e. they all found that AIM2 (absent in melanoma 2) acted as an intracellular dsDNA sensor, whose activation also leads to the formation of the inflammasome [54–57]. In contrast to other DNA sensors, such as DAI (DNA-dependent activator of IFN-regulatory factors) and one other PYHIN protein, IFI16 (interferon gamma-inducible protein 16), AIM2 does not mediate the type I IFN production [58, 59]. IFI16 is also a DNA sensor capable of assembling inflammasomes but it seems to specialize in searching for nuclear intruders, whereas AIM2 detects foreign DNA in the cytoplasm [54, 56, 57, 60, 61]. IFI16 has two HIN domains and a PYD domain, whereas AIM2 possesses only one HIN domain [62] (Fig. 3). RIG-I-like receptors (RLRs) are intracellular sensors that recognize cytosolic RNA derived either from viral infection or repeated replication [63] (Fig. 3).

## Inflammasomes become activated in AMD

Inflammasome activation in the RPE was reported for the first time in 2012 [64–66]. In all studies so far, NLRP3 has been the responsible receptor in RPE cells and it is activated by a variety of inducing agents including lipofuscin and drusen components e.g. N-retinylidene-N-retinylethanolamine (A2E) and amyloid-β [67, 68]. Fibrillar Aβ1–40 can contribute to inflammasome signaling, for example by inducing the complement activation and MAC formation [69]. The ultimate mechanism remains to be clarified but in primary human lung epithelial cells, MAC triggered NLRP3 inflammasomes by increasing the intracellular Ca^2+^ concentration with the subsequent loss of the mitochondrial transmembrane potential [70]. There are many other danger signals for NLRP3 inflammasomes in the RPE, e.g. accumulation of *Alu* RNA, the appearance of the lipid peroxidation end product HNE (4-hydroxynonenal), as well as the presence of intracellular protein aggregates accompanied by a decline in the efficiency of autophagy [9, 64, 66]. UV radiation cannot reach the adult retina but blue light (peaking at approx. 450 nm) is a potential inflammasome activator also at the retinal level [71]. A recent study revealed an interesting mechanistic link between excessive iron and AMD, showing that iron accumulation resulted in increased levels of short interspersed nuclear elements (SINEs), such as the NLRP3 agonist *Alu* RNA [64, 72]. Iron overload has been associated with the AMD-related tissue damage although the previously recognized mechanism has been linked to the induction of oxidative stress via the Fenton reaction that produces highly reactive hydroxyl radicals [73]. Furthermore, the iron-catalysed free radical-mediated production of 7-ketocholesterol (7KCh) from cholesterol has been shown to be capable of activating NLRP3 inflammasomes in the RPE [74]. Although details remain still largely sketchy, all three main mechanisms involving P2X7-dependent signaling, lysosomal destabilization, and oxidative stress have been shown to participate in the activation of NLRP3 also in the RPE-related inflammasome assembly [64–67, 75–77].

In addition to RPE, the inflammasome activation in the immune cells accumulating in the retinal area can contribute to the pathogenesis of AMD [65, 74, 78, 79]. For example, peripheral myeloid leukocytes responded by activation of the NLRP3 inflammasome after exposure to the C1q complement component and other drusen fragments extracted from the AMD eyes [65]. Mouse mononuclear cells deficient of *cx3cr1* gene autoactivated the inflammasome signaling in an ATP/P2X7-dependent manner and thereby promoted photoreceptor toxicity [78]. The oxysterol 7KCh accumulating in the choriocapillaris, Bruch’s membrane, and RPE layer induced even greater inflammasome-mediated cytokine production in microglia and macrophages than in RPE cells [74]. The exposure of microglia to sublethal concentrations of 7KCh can also lead to NLRP3 inflammasome-mediated activation and polarization of microglia towards the M1 phenotype [79]. When those cells were transplanted into the subretinal area, they were capable of promoting CNV (choroidal neovascularisation).

Although RPE and retinal inflammatory cells can produce both inflammasome-dependent cytokines, the cytokine release can be biased towards either IL-1β or IL-18. In human ARPE-19 cells, HNE stimulated the production of both cytokines, whereas treatment of the cells with the proteasome inhibitor MG-132 and the vacuolar H+ ATPase inhibitor, bafilomycin A favoured the release of IL-1β [9, 66]. Microglia and macrophages showed preferential production of IL-1β rather than IL-18 after an exposure to 7KCh, whereas in RPE cells the situation was reversed [74]. When one considers the propensity of 7KCh-treated microglia to promote CNV in the subretinal space, it could be argued that IL-1β may be involved in the pathological neovascularization process. This is in line with the evidence that IL-1β promoted the production of VEGF, whereas the release of IL-18 was inversely correlated with the amount of secreted VEGF [65, 80–83]. IL-18 has been proposed to be protective in wet AMD [65, 75, 82] but detrimental for geographic atrophy [64, 84, 85], but the overall situation needs to be fully clarified [86–89]. In therapeutic terms, one would wish to achieve a substantial inhibition of inflammasome activation. Some attempts have been made to arrest the inflammasome signaling in the RPE, e.g. by blocking the priming phase with vinpocetine, a compound that inhibits the activity of NF-κB, or by preventing pro-caspase-1 processing by administering a virally transduced CARD domain of the adaptor protein ASC [90, 91].

## Soluble PRRs

In addition to many cell-associated receptors, there are also soluble pattern recognition molecules, such as circulating complement components and pentraxins. Activation of complement triggers a cascade of protease reactions producing opsonins, membrane pore complexes, and pro-inflammatory mediators [92]. There are three different ways to induce complement activation but all of them result in the formation of a complex called C3 convertase that cleaves component C3 into C3a and C3b [93]. C3b binds to C3 convertase forming C5 convertase, which in turn cleaves the complement component C5 into C5a and C5b [92]. Components C5a and C3a are called anaphylatoxins due to their ability to promote inflammation [92]. They can attract and activate mast cells, and act directly on blood vessels to increase their permeability and induce the production of adhesion molecules [94, 95]. C5a also recruits neutrophils and monocytes to the site of inflammation and activates these cells once they are in position [93]. C3a tends to attenuate rather than inducing the LPS-induced endotoxemia activating primarily other granulocytes than neutrophils [96, 97]. In conjunction with MAC, C3a and C5a can also contribute to inflammasome signaling [98–102].

Pentraxins are evolutionarily conserved pattern recognition molecules that are often divided into two groups according to their length. Short pentraxins CRP (C-reactive protein) and SAP (serum amyloid P) are the primary acute phase proteins in humans and mice, respectively [103, 104]. Production of CRP and SAP in the liver results from the systemic consequences of the actions of several potent pro-inflammatory cytokines, such as IL-6 and IL-1. PTX-3 is an example of a long pentraxin, produced locally by different types of cells, e.g. endothelial cells, fibroblasts, adipocytes, chondrocytes, and mononuclear phagocytes, in response to various pro-inflammatory signals, such as IL-1β, TNF-α, and LPS [103, 104].

## Inheritable predisposition to AMD is strongly associated with alterations in the genes encoding complement factors

### Complement factor H polymorphism is a major genetic risk factor for AMD

Evidence emerging from recent studies has indicated that about half of the variation in the severity of AMD is explained by genetic factors [105]. Interestingly, a significant proportion of the AMD heritability is associated with the genes of the immune system, especially those coding for complement components [105]. The Y402H (Tyr402His) variant of the complement factor H is the best-known genetic risk factor for AMD [106–109]. This mutation is related to AMD susceptibility especially in Caucasians, whereas another missense mutation of CFH, I62V (Ile62Val), is more prominent in Asian populations [110]. CFH is a glycoprotein composed of 20 short consensus repeats (SCR), whose main function is to inhibit the activation of the alternative complement pathway [111].

The acute phase protein C-reactive protein (CRP) is one of the many binding partners of CFH. Previously, SCR-7 and SCR-8/11 have been proposed as being domains capable of interacting with CRP but Okemefuna et al. challenged those results when they evaluated the properties of denatured CRP protein which had been used in earlier studies [111]. When these workers used functionally active proteins, they were able to confirm that SCR-6/8 could bind to CRP, and they identified SCR-16/20 as a new domain responsible for the CRP binding. Since the Y402H substitution is located in the SCR-6/8 domain, its presence results in weaker binding properties of CFH to CRP [111, 112]. Therefore, RPE-choroid cells of homozygous Y402H AMD patients are less well protected from the increased levels of CRP. The Y402H polymorphism does not, however, affect the binding of CFH to PTX3, whose primary and secondary binding sites are SCR19 and SCR7, respectively [113]. Malondialdehyde (MDA) is another binding partner of both SCR7 and SCR20 segments in CFH. MDA is common lipid peroxidation product that forms protein adducts capable of inducing inflammation and RPE damage [114, 115]. There are at least three strands of evidence for an association between oxidative stress and complement activation in the pathogenesis of AMD (1) phagocytosized oxidized POS material can disturb the synthesis and the secretion of CFH in RPE cells, (2) the inability of the H402Y variant to generate anti-inflammatory iC3b components on MDA-loaded surfaces, (3) the finding that oxidative stress can regulate the expression of CFH and CFB [116–119]. Rohrer et al. also showed that oxidative stress predisposed RPE cells to complement-mediated injury and they later confirmed that alternative pathway of complement was needed to observe the ER stress and lipid accumulation by cigarette smoke and oxidative stress [120, 121]. By binding MDA, CFH could prevent the uptake of MDA-modified proteins by macrophages and block the induction of inflammation, but the H402Y polymorphism disturbed that binding process [115]. A chimeric mouse model was developed by expressing mutated SCR-6/8 of human CFH in the middle of murine CFH SCRs. It was found that RPE cells in these animals displayed an increased susceptibility to oxidative stress, elevated accumulation of MDA–protein adducts in the retina, higher amounts of activated microglia cells/macrophages in the subretinal space, and upregulated pro-inflammatory genes in the RPE, microglia, and macrophages [122]. Activated macrophages have also been found to be capable of regulating the expression of complement factors in RPE cells, and especially M1-type macrophages may promote the activation of the alternative pathway under inflammatory conditions [123].

### AMD-related variations in other complement factor genes

AMD-related genetic variations have also been detected in the complement factors 3 (C3), and I (CFI) [124–130]. Moreover, alterations in the gene of serpin peptidase inhibitor, clade G, member 1 (SERPING1), that regulates the activation of the complement system, have been associated with an increased risk of AMD [131]. Aging, pro-inflammatory cytokines TNF-α and IFN-γ, as well as extended exposure to POS material increase the expression of CFB in the RPE, which can promote AMD-associated neovascularization [118, 132, 133]. In combination with the accumulation of the C3 component, it has been reported that increased production of CFB by RPE cells also contributes to increased complement activation in the retina [118]. The findings that some point mutations in the C2 and CFB genes have been found protective against AMD support the hypothesis that there is an association between complement system and AMD [134–137].

The importance of complement activation has been emphasized especially in the development of wet AMD. The C3a, C5a, and MAC complexes found in subretinal drusen plaques have been linked to increased expression of VEGF and the formation of CNV [120, 138]. In addition, the production of CFB is itself sufficient to promote neovascularization, at least in the widely used animal model of wet AMD, where laser photocoagulation of RPE and Bruch’s membrane induces CNV [133]. It has also been reported that this treatment not only induces the production of VEGF and attracts leukocytes to the injured tissue but also activates the complement cascade [138]. Consistent with the observations of activation, the complement regulatory protein, CD59, a protein that prevents the MAC formation, is capable of inhibiting the CNV process [139, 140]. In addition to highlighting the role of the drusen, it has been postulated that oxidative stress-induced phospholipid-containing neoepitopes become recognized by autoantibodies, and the formation of these pathological complexes can trigger the complement activation, resulting in VEGF secretion and CNV [141].

Complement factors can promote AMD also by activating inflammosome signaling [65]. In addition to enhancing inflammation, the C3a produced by RPE cells can induce the formation of basal deposits [142]. Amyloid-β which can be found in the drusen is capable of harnessing recruited macrophages and microglia to produce cytokines that induce CFB formation in the RPE [143]. The promotion of pro-inflammatory environment is also involved in the pathological effects of cigarette smoke when it induces C3a and C3b, especially in the absence of Nrf2 [144]. By regulating the production of IL-1β and IL-6, C3a and C5a can also promote Th17 differentiation and IL-17 production, which have recently emerged as potential players in adaptive immunity in the pathology of both wet and dry AMD [98, 145–149].

### AMD-related genetic variation in the immune system is not entirely restricted to complement factor genes

There are other central immune system components associated with the genetic susceptibility to AMD e.g. the chemokine (fractalkine) receptor CX3CR1 and chemokine CCL-2 (C–C motif ligand 2; monocyte chemotactic protein 1, MCP-1). CX3CR1 is a double-edged sword—it can confer protection or cause destruction, depending on the tissue and pathophysiologic conditions [150]. There is convincing evidence suggesting that the normal function of CX3CR1 would be to protect from AMD rather than to cause the disease [151–155]. The protective role of CX3CR1 might result from its supportive functions, such as the regulation of retinal microglia and its tendency to diminish the expression of CCL2 and thereby the recruitment of pro-inflammatory CCR2^+^ monocytes to the retina [155, 156]. *Cx3cr1*^*GFP/GFP*^ murine monocytes were shown to contribute to photoreceptor degeneration by stimulating the autonomous activation of P2X7 receptors and IL-1β secretion through spontaneous ATP release [78]. These findings suggest that CX3CR1 would play a significant role in maintaining tissue homeostasis, a process which has been termed as parainflammation [157, 158]. Genetic variants V249I (Val241Ile) and T280M (Thr280Met) of CX3CR alter the binding of fractalkine by circulating leukocytes and along with other age-related diseases, this defect has been associated with the development of AMD [151, 159–162]. There are also contradictory results, i.e. no evidence for altered function of CX3CR1 in the pathogenesis of AMD [163–165]. A number of association studies have been performed using double knock-out mice lacking both *Cx3cr1* and *Ccl2* genes [152–155, 163] but their findings have been questioned since many transgenic mice carry the *rd8* (retinal degeneration 8) mutation in their *Crb*-*1* (crumbs-like 1) gene that also results in retinal degeneration [164, 166, 167]. Subsequent studies have found contradictory results when using mice that do not carry the *Crb*-*1/rd8* mutation, although they have provided further evidence that mice with the *rd8* background still develop an RPE-related pathology reminiscent of AMD [154, 163, 164, 168, 169]. However, in a recent pooled analysis from five prospective human studies, no unambiguous association could be detected between common CX3CR1 variants and AMD [165]. Instead, the effect of CX3CR1 variants was found to depend on several factors, such as diet, obesity, and the presence of predisposing variants of the complement components [165]. This conclusion is not surprising in view of the well-known multifactorial nature of AMD pathogenesis.

In addition to the double-knockout mouse model, there are also mice lacking only the *Ccl2* gene. Several studies performed using those animals, have indicated that the absence of CCL2 evokes changes typical of AMD, and this could also be an indication of a failed parainflammatory response [170, 171]. However, a study conducted with AMD patients and control subjects from The Netherlands and the US, detected no associations between CCL2, CCR2, or TLR4 and AMD [172].

## Inflammatory response

**Fig. 5:**
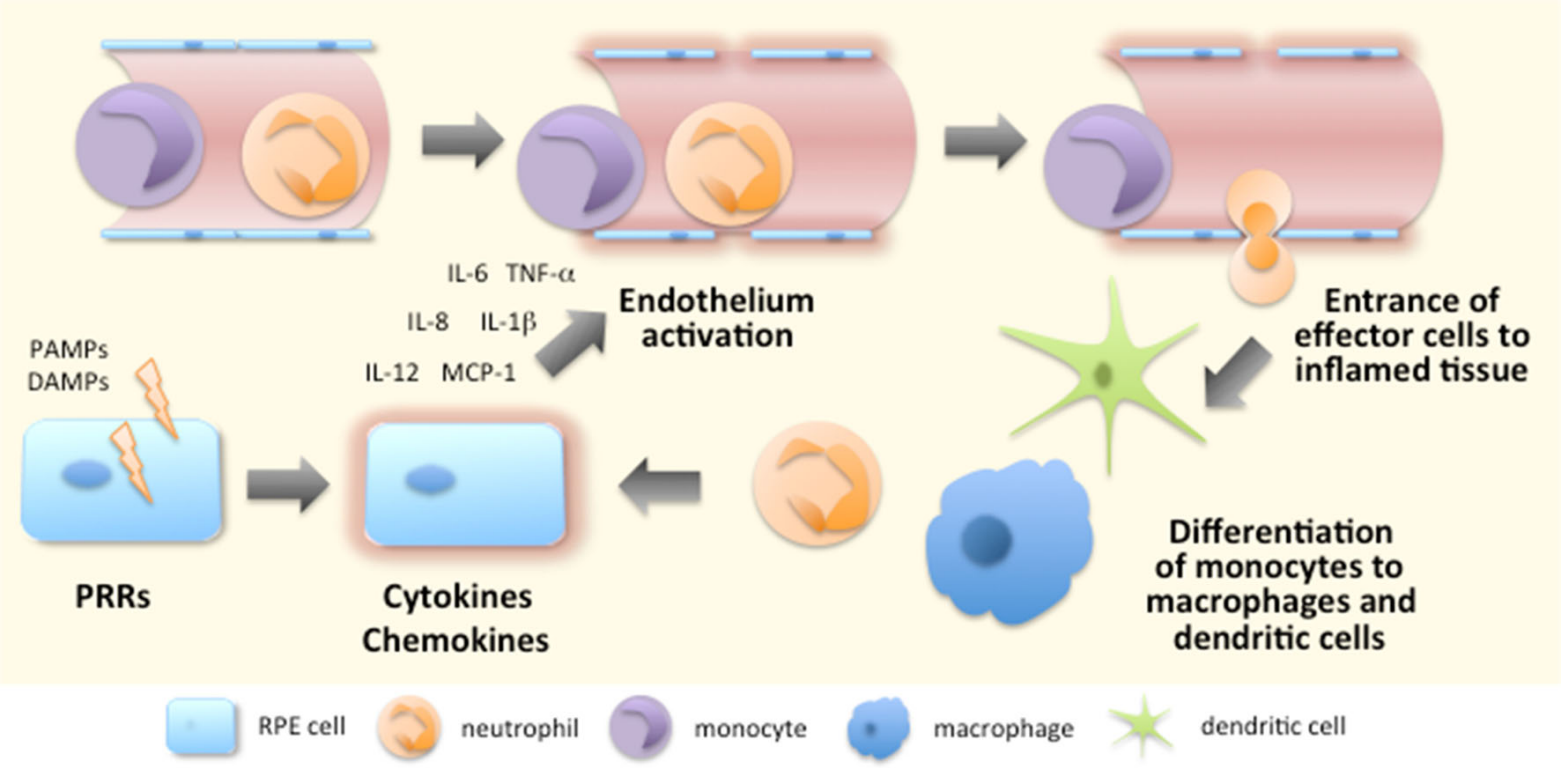
Initiation of the inflammatory response. Recognition of PAMPs and DAMPs by PRRs triggers intracellular signaling resulting in the production of pro-inflammatory cytokines and chemokines. The released mediators contribute to the activation of endothelium, e.g. elevated expression of adhesion molecules and increased vascular permeabilization. Circulating leukocytes interact with adhesion molecules expressed by endothelium, slow down their speed and start rolling along the endothelial layer. The chemokine gradient which originates from the inflamed tissue becomes sensed by leukocytes that start expressing integrins to permit their tighter binding to endothelial cells. Finally, leukocytes leave the circulation to seek out the inflamed tissue where monocytes differentiate into macrophages and dendritic cells according to the local conditions

Activation of their PRRs causes cells to secrete cytokines and chemokines, e.g. IL-1β, IL-6, TNF-α, IL-12, and IL-8 [CXCL8; chemokine (C-X-C motif) ligand 8], to which other cells respond (Fig. 5). The local effects of IL-1β and TNF-α include the activation of endothelial cells, which is one of the most prominent processes at the beginning of inflammation [173]. Endothelial cell activation is characterized by increased expression of leukocyte adhesion molecules, cytokines, growth factors, and HLA molecules [174, 175]. Moreover, in order to prevent the spreading of a potential pathogen, their phenotype changes from antithrombotic to prothrombotic [174]. Subsequently, the permeability of blood vessels increases and circulating leukocytes begin to make contact with the adhesion molecules expressed by endothelial cells [175] (Fig. 5). Leukocytes reach their target location at the site of inflammation by following the increasing chemokine gradient, finally leaving the circulation and moving towards the damaged tissue [176] (Fig. 5). It is not only white blood cells that enter the inflamed tissue but also fluids and various plasma proteins gain access to these sites of tissue damage [175]. Together they induce the typical signs of inflammation, i.e. rubor (redness), tumor (swelling), calor (heat), and dolor (pain) [177]. Later, functio laesa (impaired function) and fluor (secretion) have also been suggested as being other characteristics of inflammation [177, 178].

IL-8 or CXCL8 was the first chemokine to be characterized; this compound attracts neutrophilic granulocytes that are the first effector cells to reach the site of inflammation [173, 179, 180] (Fig. 5). Other CXC chemokines, such as KC (keratinocyte-derived chemokine; CXCL1) and MIP-2 (CXCL2) also participate in the recruitment of neutrophils [180]. Neutrophils are efficient phagocytes and important in destroying microbes if they are the cause of the acute inflammation. Activated neutrophils kill pathogens in several ways (1) by producing reactive oxygen species, (2) by releasing active peptides, and (3) by forming extracellular fibers called neutrophil extracellular traps (NETs) through the release of granule proteins and chromatin [181, 182]. NETs not only bind microbes, preventing them from spreading and ensuring that there are high local concentrations of antimicrobial agents but these fibers can also promote adaptive immunity and function even in sterile inflammation [181, 183]. It is this active interaction with other immune cells that broadens the significance of neutrophils in innate and adaptive immunity [184]. Neutrophils also regulate angiogenesis by producing VEGF [180, 185].

Monocytes follow neutrophils to inflammatory foci and once embedded in the tissues, they differentiate to macrophages or dendritic cells depending on local conditions with cytokines, growth factors, and possible microbial components [186, 187] (Fig. 5). Macrophages and dendritic cells are efficient antigen-presenting cells (APCs) that can internalize particulate antigens e.g. derived from pathogens or dying cells [188, 189]. After binding the antigen, cells migrate from inflamed tissue to local lymph nodes where they present it to other cells of the immune system and TNF-α is involved in promoting the transition of these antigen-presenting cells [188, 189]. The cells of adaptive immunity assist innate immune cells in coping with the inflammation but also make the responses more specific in order to prevent collateral damage to healthy cells in the vicinity of the inflamed tissue [190].

Macrophages are very flexible cells changing their phenotype and functions depending on the environment in which they find themselves [191]. An inflammatory environment favors M1 macrophages that produce high levels of pro-inflammatory cytokines, such as (pro)IL-1β, TNF-α, IL-6, IL-12, as well as inducible nitric oxide synthase (iNOS) leading to the Th1-type immune response [192, 193]. The so-called classically activated M1 macrophages become activated by IFN-γ and TNF-α. IFN-γ can be produced by natural killer (NK) cells during innate immune responses, and by T helper 1 (Th1) and cytotoxic CD8^+^ T lymphocytes during adaptive immune responses, whereas antigen-presenting cells (APCs), including macrophages themselves, are efficient in producing TNF-α [191, 192].

Th2-type cytokines IL-4 and IL-13 are direct activators of the M2 macrophages [194]. Those cytokines can be secreted by many different cell types including innate and adaptive immune cells, epithelial cells, and tumor cells. In addition to playing important roles in physiological events, such as homeostasis, wound healing, and tissue repair, the actions of M2 macrophages have been implicated in pathological processes, such as inflammation, hypersensitivity, or choroidal neovascularization [191, 194, 195]. However, the inflammation associated with M2 macrophages is not as intense as that induced by their M1 counterparts. For example, M2 macrophages are inefficient in antigen presentation, and they have rather poor capabilities for eliminating intracellular pathogens, nor do they evoke the production of Th1-type proinflammatory cytokines or toxic oxygen and/or nitrogen radicals [196]. M2 macrophages are also poor at dealing with infections caused by intracellular pathogens [191]. Moreover, while the propensity of M2 macrophages to secrete extracellular matrix components certainly helps in wound healing, in chronic conditions, it also predisposes to pathological fibrosis [191, 194]. In addition to neutrophils, the chemokines released by M2 macrophages attract and activate also other granulocytes, i.e. basophils, eosinophils, and mast cells. These cells are known to participate in the typical Th2-type responses; i.e. the beneficial actions, e.g. combatting parasite infections but also in detrimental effects, such as evoking allergies and hypersensitivity reactions [194].

In addition to distributing the subdivision into M1 and M2 cells, there is a third functional class of macrophages—so-called regulatory macrophages, which have been classified as a subgroup of M2 macrophages [196]. Similar to the M1 cells, regulatory macrophages can produce high levels of nitric oxide (NO), express the co-stimulatory molecule CD86, and present antigens to T lymphocytes [196]. However, regulatory macrophages promote the Th2-type response by producing high amounts of IL-10, whereas M1 cells favor Th1-type reactions by releasing IL-12 [196]. IL-10 is an anti-inflammatory cytokine and therefore, regulatory macrophages are thought to attenuate inflammation [191]. A great many different signals, such as immune complexes of antibodies and soluble antigens, prostaglandins, glucocorticoids, apoptotic cells, and IL-10, can contribute to the activation of regulatory macrophages [191]. In addition to priming, a subsequent signal, e.g. mediated through a TLR is needed for their full activation [191].

## Aging induces changes in the immune system

Immunosenescence is a term used to describe altered immune functions during aging. Despite the apparent slowdown of many functions, the term dysfunction with respect to immunosenescence is somewhat misleading. Instead of a total loss of the function, aging alters the functions of the immune system so that it no longer resembles the immune system of the young individuals. Simultaneously with a reduction in the naïve T cell pool, there is an increase in the numbers of memory T cells, especially those of CD8+ T cells that have lost their CD80 and CD86-binding co-stimulatory molecule CD28 [197, 198]. The increased memory T cell numbers have been postulated to result from an attempt to maintain the cell count in balance, but this may lead to the exhaustion of remaining T lymphocytes with limited replicative capacity [199]. The loss of CD28 expression is accompanied by an age-dependent de novo induction of prototypic NK cell receptor CD56 on non-dividing senescent T cells [200].

In addition to quantitative and qualitative changes appearing in T cells, age-related modifications in the B cell pool contribute to unsuccessful vaccination responses, as well as to the increased frequency and greater severity of infections [198]. Other unfavorable changes include decreased amounts of mature human B cells, diminished reactivity to T cell-dependent antigens, and a deficiency in class switch recombination.

The functions of the major innate immune effector cells, such as neutrophils, monocytes, macrophages, and dendritic cells also undergo age-related modifications. Those include changes in the PRR expression, aberrant signaling and disturbed cytokine production, as well as decreased migration, phagocytosis, and killing of ingested micro-organisms [201]. For example, the diminished capacity of neutrophils to phagocytize pathological particles and the failure to induce a respiratory burst to destroy ingested material accompanied by an inability to undergo apoptosis can contribute to prolonged inflammation. Furthermore, it is known that the clearance of apoptotic cells by macrophages is diminished [202].

## Inflammation is clearly present in the AMD pathology

Increased oxidative stress, reduced proteostasis, and ever-increasing dysfunctionality are just some of the stress factors that can induce inflammation in aged RPE cells. The concurrent attenuation of protective mechanisms, e.g. antioxidant responses and DNA repair systems, further amplify the destructive effects and promote the conversion of what should be a protective response into a chronic and deleterious pathological process.

### Drusen serve as inflammatory nodes in the pathogenesis of AMD

RPE cells are the origin of numerous components found in drusen deposits; in conjunction with pigment mottling, these are the first clinical signs detectable in the AMD [25, 203–205]. Subretinal drusen resemble the extracellular deposits found in Alzheimer’s disease, amyloidosis, atherosclerosis, elastosis, and dense deposit disease [38, 206, 207]. Drusen are known to contain many potentially damaging constituents including lipids, lipoproteins, RPE-derived cellular debris, e.g. organelles, melanin granules, and lipofuscin, amyloid-β, apolipoprotein E (APOE), clusterin, serum albumin, crystallin, tissue metalloproteinase inhibitor 3 (TIMP-3), and oxidation by-products, as well as numerous inflammation-related factors, such as complement components, immunoglobulins, HLA molecules, and acute phase proteins like vitronectin, fibrinogen, α1-antichymotrypsin, and pentraxins [208–218]. Elevated oxidative processes, stressed autophagy, and increased exo- and transcytosis in RPE cells have been associated with the formation of drusen between the RPE and the choroid layers [219, 220]. Moreover, there is solid evidence suggesting that chronic low-level inflammation and complement activation play major roles in the formation of drusen [212, 217, 221–226]. Isolated drusen material has also been proven to be pro-inflammatory through the activation of both traditional and the more recently discovered signaling systems, such as NF-κB and the inflammasome pathways, respectively [65, 68, 90, 227].

### Leukocytes contribute to the pathogenesis of AMD

Retinal microglia and recruited macrophages play an important role in parainflammation, i.e. the maintenance of tissue homeostasis and the clearance of debris from the subretinal space [158, 228, 229]. Aging induces changes in the immune system, which also alters the function of leukocytes. For example, the increased activity of matrix metalloproteases (MMPs) enhances the cleavage of FasL on the cell surfaces resulting in a limited apoptosis of invading inflammatory cells [195, 230–233]. Soluble FasL also recruits M2-type macrophages that promote neovascularization [195, 234]. In a healthy eye, M2 macrophages in particular confer protection from degenerative changes but in AMD, also the proportion of pro-inflammatory M1 macrophages increases and the stress becomes overwhelming [235, 236]. After disrupting the homeostasis of the eye, the accumulation of immune cells causes more harm than benefit. The altered conditions may also change the effects of cytokines depending on the stimulant. For example, Wu et al. have demonstrated how the anti-inflammatory cytokine, IL-10, can inhibit M1 but not M2 macrophage-derived VEGF production in a context-dependent manner [237].

Although normally associated with healthy aging, an inflammatory environment also alters the functionality of senescent T cells. Increased numbers of CD56^+^ T cells have been detected in the blood of AMD patients when compared to aged control subjects [238]. Elevated numbers of CD56^+^ lymphocytes have been associated with many autoimmune diseases, such as rheumatoid arthritis, Behçet’s uveitis, psoriasis, and systemic lupus erythematosus [239–242]. Regardless of the numerous autoimmunity-related markers, such as anti-retinal and anti-RPE autoantibodies and diverse contributions of IL-17, AMD cannot simply be designated as an autoimmune disease [145, 146, 243–248]. Changes in the CD56^+^ T cell levels do not only occur in autoimmune disorders but have also been detected, e.g. in the coronary artery disease, a condition that shares various risk factors and biomarkers with AMD and may even predispose to the disease [249, 250].

### Systemic inflammatory biomarkers of AMD

The multitude of inflammation-related plasma proteins in the drusen refers to the involvement of systemic immunological processes in the pathogenesis of AMD. Some research has been conducted with urine samples [251] but most putative biomarkers have been investigated in peripheral blood, serum, or plasma. For example, increased levels of complement components have been assayed in the blood of AMD patients [252–255]. Elevated levels of regulatory proteins, such as CD21 (complement receptor 2), CD35 (complement receptor 1), CD46 (membrane cofactor protein, MCP), CD55 (decay-accelerating factor, DAF), or CD59 (protectin), may resemble increased complement activity but a significantly lower expression can be evidence of dysregulated control [256, 257]. Instead, the lack of association between AMD and SNPs in CFP (properdin), CD46, CD55, and CD59 suggests that the gene variants of those regulatory proteins do not increase an individual’s susceptibility to AMD [258]. In contrast, AMD patients with the homozygous CC variant of the Y402H substitution in CFH displayed higher systemic concentrations of central pro-inflammatory cytokines IL-6 and TNF-α when compared to heterozygous CT or non-risk TT variants [259]. Both of these cytokines can promote pathological changes in the RPE [260–262]. TNF-α also reflects the activity of T lymphocytes and macrophages that are known to be associated with the pathological changes of AMD [263–267]. In particular, macrophage-derived TNF-α and IL-1β might serve as biomarkers for choroidal neovascularization [263, 264]. In addition, macrophage activation may also result in the release of MMPs, and increased plasma levels of these enzymes have been detected in AMD patients [268]. The association between elevated systemic IL-6 levels and AMD has been supported by several other studies [269–271] although contrasting results have also been published [272].

The acute phase protein, CRP, has been one of the most widely studied putative blood biomarkes for AMD. Despite the somewhat inconsistent findings, one meta-analysis conducted by Hong et al. in 2011 from 11 studies (nine cross-sectional and two prospective) with almost 42,000 participants revealed that those subjects with serum levels of CRP higher than 3 mg/l had a twofold higher likelihood of late AMD in comparison to those subjects having CRP levels lower than 1 mg/l [273]. The pooled analysis of five large prospective nested case–control studies reported by Mitta et al. in 2013 supported the view that elevated serum CRP levels could be associated with AMD [274]. In a recent study with over 5000 aged British subjects, higher serum CRP levels were associated with increased risk of AMD in the longitudinal, but not in the cross-sectional analysis [275]. There was a modest association between high CRP levels and the 20-year cumulative risk for early AMD in the Beaver Dam Eye Study with almost 6000 participants [271].

Significant and moderate increases in the plasma concentrations of inflammasome-related cytokines IL-18 and IL-1β, respectively, in patients carrying the high risk CC alleles of Y402H variant raises an intriguing possibility that there is systemic or continuous inflammasome activation in patients suffering from dry AMD [259]. In addition to those factors mentioned above, there are many other inflammation-related factors, such as eotaxin, fibrinogen, IP-10, long pentraxin 3, sFasL (soluble Fas ligand), sICAM-1 (soluble intercellular adhesion molecule-1), sTNFRII (TNF-a receptor II), that have also been proposed as biomarkers of AMD [267, 272, 276–280]. Although there has been increased research in obtaining reliable biomarkers for AMD, no selective blood biomarker has been found that meets the requirements of early AMD detection. The pro-inflammatory environment may, however, nudge cellular immunity towards a pathological phenotype with these changes becoming visible as the subject reaches an advanced age.

## Summary

Inflammation is a cellular defence mechanism, in which foreign or damaged material becomes sensed by various PRRs [28]. The ligand recognition process triggers the activation of intracellular signaling pathways resulting in the production of numerous pro-inflammatory mediators [34]. The activated endothelium in the blood vessels promotes and attracts effector cells and there is an accumulation of soluble proteins within inflamed tissue [174, 175]. Effector leukocytes, such as granulocytes, monocyte-derived macrophages and dendritic cells, as well as lymphocytes utilize a multitude of mechanisms for meeting the challenge of restoring the tissue homeostasis [181, 183, 184, 188–194, 196].

AMD is an ocular disease with inflammation strongly interwoven into its pathogenesis. Several PRRs become activated by endogenous intra- and extracellular danger signals inducing an inflammatory response beyond the homeostasis-maintaining para-inflammation. Degenerative changes in RPE cells trigger a vicious circle that promotes the development of chronic inflammation in the retina and the choroid. Age-related changes in the immune system contribute to this destructive process by altering the functions of immune cells. Currently, there is no cure to AMD, and changes at the cellular level are already significant when the first symptoms appear. Various local and systemic inflammatory molecules have been proposed as being biomarkers of AMD but at present, no specific and reliable marker has been found. If there were a selective marker, this would help in the initial clinical diagnosis, preferably before the disease had progressed to a symptomatic phase. Moreover, biomarkers could also help to clarify the mechanisms behind AMD as well as helping to monitor the response to therapy.
